# Comprehensive evaluation of Dragon’s Blood in combination with borneol in ameliorating ischemic/reperfusion brain injury using RNA sequencing, metabolomics, and 16S rRNA sequencing

**DOI:** 10.3389/fphar.2024.1372449

**Published:** 2024-05-09

**Authors:** Jiahui Ren, Xue Zhang, Lingjuan Zhou, Wanyu Cao, Lixia Zhang, Xi Chen, Guang Li

**Affiliations:** ^1^ Institute of Medicinal Plant Development, Chinese Academy of Medical Sciences & Peking Union Medical College, Beijing, China; ^2^ Yunnan Branch, Institute of Medicinal Plant Development, Chinese Academy of Medical Sciences & Peking Union Medical College, Jinghong, China; ^3^ Yunnan Key Laboratory of Southern Medicine Utilization, Jinghong, China; ^4^ Heilongjiang University of Chinese Medicine, Harbin, China; ^5^ Xishuangbanna Dai Autonomous Prefecture People’s Hospital, Jinghong, China

**Keywords:** ischemic stroke, dragon’s blood, borneol, RNA-sequencing, metabolomics, intestinal flora

## Abstract

Ischemia/reperfusion (IR) can induce deleterious responses such as apoptosis, inflammation, and oxidative stress; however, there are currently no efficient therapeutics to treat IR brain injury. Dragon’s blood (DB) plays a significant role in treating ischemic stroke in China. Borneol (B) is an upper ushering drug that guides drugs to the target organs, including the brain. Therefore, we hypothesized that the combination of DB and B (DB + B) would provide cooperative therapeutic benefits for IR brain injury. To confirm this, we first investigated the protective effect of DB + B in an IR brain injury rat model using the modified neurological severity score (mNSS), infarction size measure, HE staining, and laser speckle contrast imaging. Then, we comprehensively evaluated the mechanism of DB + B in ameliorating IR brain injury based on RNA sequencing, serum untargeted metabolomics, and 16S rRNA sequencing. We have confirmed that DB + B enhanced the efficacy of the ischemic stroke treatment compared to DB or B alone for the first time. Our study provisionally confirms that the mechanism by which DB + B prevents IR brain injury is related to the maintenance of intestinal microecological balance and regulation of metabolic dysfunction, thereby suppressing inflammation and regulating immunity. DB + B may effectively regulate intestinal flora including o_Pseudomonadales, s_*Bacteroides*_caecimuris, o_unidentified_Bacilli, f-Pseudomonadaceae, and g-Pseudomonas, mainly regulate serum metabolites including improve the protective benefit of IR brain injury lysoPCs and lysoPEs, thus inhibiting TLR4/MyD88/NF-κB and IL-17 signing pathway to reduce inflammatory reactions. hat this mechanism is associated with the maintenance of intestinal flora balance and the regulation of metabolic dysfunction, thereby suppressing inflammation and regulating immunity. This provides scientific support for the clinical translation of DB + B in the prevention and treatment of ischemic stroke and establishes a basis for further investigation of its therapeutic mechanism.

## 1 Introduction

Stroke is characterized by high morbidity and mortality rates leading to long-term disability (Hollist et al., 2021). Stroke is mainly divided into hemorrhagic stroke and ischemic stroke, the latter of which is caused by sudden blockage of the internal carotid artery or middle cerebral artery and accounts for 60%–80% of stroke cases (He et al., 2021). Currently, restoration of blood supply (reperfusion) through recombinant tissue-type plasminogen activators remains the only treatment strategy for ischemic stroke (Hu et al., 2018).

Ischemic stroke is characterized by neuronal dysfunction and death as a result of both ischemic and reperfusion injury to the brain (Peng et al., 2022). As a complex pathophysiological process, ischemia/reperfusion (IR) brain injury is associated with targeting excitotoxicity, calcium overload, oxidative stress, autophagy, inflammation, apoptosis, and blood-brain barrier damage (Chamorro et al., 2016; Cheon et al., 2018). However, because of its narrow therapeutic window and unavoidable revascularization, IR brain injury currently does not have an efficient therapeutic approach. Therefore, new therapeutic alternatives for cerebral ischemic stroke are urgently needed.

Metabolic abnormalities are important factors that increase the susceptibility to diseases. Ischemic stroke can cause changes in a broad spectrum of metabolites and is influenced by metabolic changes in the internal biochemical environment due to the impairment of the blood-brain barrier, consequently resulting in the aggravation of brain damage (Li et al., 2022; Wang et al., 2022). The differential metabolites in the serum between the treatment and control groups are often potential biomarkers of disease, so serum untargeted metabolomics has been used to distinguish the differential metabolites (Montaner et al., 2020).

The intestinal flora, which produces numerous metabolites that influence the host, plays an important role in modulating brain function by regulating endocrine and neurological signaling, improving immunity, and modifying drug action and metabolism (Fan and Pedersen, 2021). Disordered intestinal flora can lead to neurological disorders. The gut-brain axis has received a lot of attention in recent years. Previous research has indicated that drugs have interactions with intestinal flora (Zhang R. et al., 2020; Liu et al., 2023). Furthermore, the physiological activities of some metabolites associated with intestinal flora have attracted considerable attention, such as bile acids, amino acids, short-chain fatty acids, and tryptophan metabolites (Haase et al., 2020). Therefore, investigating the relationship between drugs and the intestinal flora is important to further study the pharmacology of drugs for ischemic stroke.

Traditional Chinese Medicine (TCM) has long played a significant role in the prevention and treatment of ischemic stroke, owing to its multi-metabolite and multi-target properties. Dragon’s Blood (DB), the red resin obtained from *Dracaena cochinchinensis* (Lour.) Chen has been shown to have a significant role in the clinical therapy of blood stasis in China (Xin et al., 2011). Its phenolic metabolites (Longxuetongluo Capsule) were authorized to treat ischemic stroke in the clinic (Pan et al., 2021). DB has been shown to inhibit platelet aggregation (Xin et al., 2011), attenuate apoptosis (Ran et al., 2016), exert anti-inflammatory and antioxidant effects (Ran et al., 2014), reduce microglial activation (Xu et al., 2022), and inhibit atherosclerosis in previous studies (Zheng et al., 2016). However, among phenolic metabolites with primary distribution in the liver and kidney, only cinnabarine and pterostilbene are preferentially distributed in the brain (Sun et al., 2018). Therefore, it is important to improve the bioavailability and brain tissue targeting of DB.

In TCM theory, Borneol (B) is an upper ushering drug that guides drugs to target organs, such as the brain. B is a natural product from plants, including *Cinnamonum camphora* (L.) J. Presl, *Dryobalanops aromatica* C.F.Gaertn., *Blumea balsamifera* (L.) DC., *Dipterocarpus turbinatus* C.F.Gaertn., *Salvia officinalis* L., *Rosmarinus officinalis* L., and *Valeriana officinalis* L. (Kulkarni et al., 2021). Several TCM preparations used in the clinical treatment of stroke incorporate B, such as Angong Niuhuang pills and Xingnaojing injections (Guo et al., 2014; Zhang Y.-M. et al., 2020). In addition, B can enhance both the oral bioavailability and brain penetration of neuroprotective drugs such as chuanxiong (Yu et al., 2021), gastrodin (Cai et al., 2008), puerarin (Yi et al., 2017), kaempferol (Zhang et al., 2015), and vinpocetine (Ma et al., 2020). Its mechanism is related to the regulation of P-glycoprotein and 5-HT expression, the ultrastructure of brain tissues, and the expression of Mdr1a, Mdr1b, and Mrp1 (Yu et al., 2013; Fan et al., 2015; Liu et al., 2021). In addition, B also has preventive and therapeutic effects against ischemic stroke (Li et al., 2021).

According to TCM theory, DB and B may be used together to improve efficacy in the treatment of ischemic stroke, but further research is required to verify this idea. Therefore, we hypothesized that the combination of DB and B (DB + B) would provide cooperative therapeutic benefits in ischemic stroke. This study investigated the protective benefits of DB + B in a rat model of cerebral IR brain injury. Then, RNA sequencing, serum untargeted metabolomics, and 16S rRNA amplicon sequencing were used to comprehensively evaluate the mechanism of DB + B in ameliorating IR brain injury. Summarizing the mechanism of DB + B in IR brain injury, we hope to contribute a guideline for further basic and clinical studies.

## 2 Materials and methods

### 2.1 Animals

All male Sprague-Dawley rats (220 ± 10 g) were obtained from Si Pei Fu (Beijing) Biotechnology Co., Ltd. (Beijing, China) and they were housed under the conditions of controlled room temperature of around 25 °C and humidity at 55% ± 10%, with a 12-h light/dark cycle. The food and water were available to access unlimitedly. All animal experiments were ethically approved by the Animal Ethics Committee of the Yunnan Branch of the Institute of Medicinal Plant Development, Chinese Academy of Medical Sciences and Peking Union Medical College and followed the Compilation of Group Standards and Implementation Guidelines of the Chinese Society for Laboratory Animals.

### 2.2 Transient middle cerebral artery occlusion (tMCAO) model

Based on the method described by Longa et al. (1989), we used a tMCAO rat model to induce focal cerebral IR brain injury. After anes-thetizing the rats with isoflurane, the common and external carotid arteries were ligated on the right side of the rats, and the internal carotid artery was embolized from the common carotid artery using a threaded bolt (RWD Life Science Co., Ltd., China). After 1.5 h of embolization, the threaded bolt was withdrawn until the black marker point protruded through the skin and was cut off. The sham rats performed similarly to the model rats except for the embolization of the middle cerebral artery with a threaded bolt.

The experimental groups were the sham group (10 mL/kg, 0.5% CMC-Na), model group (10 mL/kg, 0.5% CMC-Na), B group (10 mL/kg, 90 mg/kg), DB group (540 mg/kg), different doses of DB + B group (10 mL/kg, DB, 270 mg/kg, 540 mg/kg; B, 30 mg/kg, 60 mg/kg, and 90 mg/kg), and nimodipine group (10 mL/kg, 10 mg/kg). DB and B were synthesized by Xishuangbanna Kanglong Pharmaceutical Co., Ltd., and nimodipine was synthesized by YABAO Pharmaceutical Co., Ltd. All groups were orally administered the therapy once daily for 7 days. On the seventh day, the tMCAO model was established after 1 h of administration.

### 2.3 Modified neurological severity score (mNSS)

After 22 h of IR, neurological deficits were assessed via mNSS by four people blinded to the experimental groups (Chen et al., 1996; Chen et al., 2001). The set of mNSS is listed in Supplementary Table S1. Briefly, neurologic deficits of rats can be graded according to score: 1–6 points indicated mild injury, 7–12 points indicated moderate injury, and 13–18 points indicated severe injury.

### 2.4 Infarction size measure

Rats were sacrificed after blood was collected from the abdominal aorta. Coronal sections, 2 mm thick, were cut through the cerebrum and subsequently incubated in a solution of 2,3,5-triphenyl tetrazolium chloride (TTC, J&K Scientific, China) for 15 min at 37 °C. Brain sections were fixed in 4% paraformaldehyde overnight. All sections were photographed and measured using ImageJ software to delineate the infarct.

### 2.5 Hematoxylin-eosin (HE) staining

The brain sections were dehydrated with a 15% sucrose solution for 2 h and then with a 30% sucrose solution until they sank to the bottom. These brain sections were then cut into frozen sections (CRYOSTAR NX50, Thermo Fisher Scientific) and subjected to HE staining for microscopic examination (DP43, Olympus).

### 2.6 Laser speckle contrast imaging

Post-reperfusion cerebral blood flow was assessed using a laser speckle contrast imager (PeriCam PSI, PERIMED, Sweden), which provided high-resolution real-time blood flow images. The rats were anesthetized with isoflurane, and their fur was cut to expose the skull. Normal saline was administered to prevent skull dryness. Continually 1-min Speckle imaging (250 frames, 10s/frame) was obtained at 0.1 Hz by PIMSoft 1.11.2 software at baseline, after middle cerebral artery occlusion, after post-reperfusion, and after 24 h of post-reperfusion.

### 2.7 RNA sequencing analysis

We selected the cerebral cortex of the ischemic hemisphere of the sham, tMCAO, and DB + B groups to sequence all samples using the DNBSEQ high-throughput sequencing service (Beijing Genomics Institution, China). We used the Beijing Genomics Institution’s Dr. Tom platform to perform differential gene, Gene Ontology (GO), and Kyoto Encyclopedia of Genes and Genomes (KEGG) enrichment analyses.

### 2.8 Metabolomics analysis

Take 200 μL of the serum, add 800 μL of pre-cooled acetonitrile (at 4 °C), vortex for 10 s, leave to stand for 10 min at 4 °C, and then centrifuge at 4°C and 13,000 revolutions per minute for 15 min. Take 800 μL of the supernatant, dry with the Vacuum Centrifuge Concentrator, add 400 μL of 50% precooled acetonitrile to reconstitute, vortex for 10 s, centrifuge at 4 °C and 13,000 revolutions per minute for 15 min. Finally, extract the supernatant to obtain results. Quality control samples were prepared using equal amounts of the supernatant from each sample.

Online LC-MS analysis was conducted utilizing an Ultrahigh-performance Liquid Chromatograph (UPLC, Shimadzu Nexera Series) combined with a Quadrupole Time-of-Flight Mass Spectrometry (Q-TOF/MS, AB SCIEX X500B QTOF). Using the electric spray positive and negative ion modes with an ESI ion source. Nitrogen was utilized as the curtain gas (CUR), ion source gas 1 (GS1), ion source gas 2 (GS2), and collision gas (CAD). The MS parameters are listed in Table 1. The primary mass spectrum was scanned from 60 to 1,250 Da. The collection of primary and secondary MS data was based on information-dependent acquisition, and the 12 highest peaks with a response value of above 100 cps were selected to scan the secondary mass spectrum. The secondary mass spectrum was scanned from 50 to 1,250 Da.

**TABLE 1:** The MS parameters.

Parameter	Value (ESI+)	Value (ESI-)
Ion source voltage	4500 V	−4500 V
Ion source temperature	550 °C	550 °C
CUR	35 PSI	35 PSI
Gas1	60 PSI	60 PSI
Gas2	60 PSI	60 PSI
Declustering potential	80 V	−80 V
Collision energy	40 ± 20 V	−40 ± 20 V

The column was T3 with column temperature at 35 °C. The mobile phase A was water with 0.1% formic acid while the mobile phase B was acetonitrile with 0.1% formic acid. The gradient elution sequences are listed in Table 2. The injection volume is 5 μL at a flow rate of 0.3 mL per minute.

**TABLE 2:** The gradient elution sequence.

Time (min)	A%	B%
2.0	95.0	5.0
3.0	65.0	35.0
8.0	45.0	55.0
13.0	17.5	82.5
14.0	5.0	95.0
17.0	5.0	95.0
17.1	95.0	5.0
21.0	95.0	5.0

### 2.9 16S rRNA amplicon sequencing analysis

The intestinal contents of rats were collected aseptically. The samples were then sequenced using Metware Biotechnology Co., Ltd. for 16s rRNA amplicon sequencing. According to a previous report, the total genomic DNA of the microbiome was extracted using the cetyltrimethylammonium bromide method. 16S rRNA/18S rRNA/ITS genes of distinct regions were amplified with 15 L of Phusion^®^ High-Fidelity PCR Master Mix (New England Biolabs). A mixture of the PCR products was then purified. Sequencing libraries were generated usingTruSeq^®^ DNA PCR-Free Sample Preparation Kit (Illumina, USA). The quality of the library was evaluated using a Qubit@ 2.0 Fluorometer (Thermo Scientific) and an Agilent Bioanalyzer 2,100 system. Then, the library was sequenced on an Illumina NovaSeq platform, generating 250 bp paired-end reads. Finally, the analysis of the data was accomplished using the Metware Cloud platform (https://cloud.metware.cn).

### 2.10 Statistical analysis

All data were used GraphPad Prism 9 software for statistical analysis and presented as mean ± SEM (n ≥ 3). The comparison was analyzed using the t-test, one-way ANOVA, Mann-Whitney U test, or Wilcox test. *p* < 0.05, the indication of statistical significance.

## 3 Results

### 3.1 DB + B alleviates IR brain injury in tMCAO rats

**FIGURE 1:**
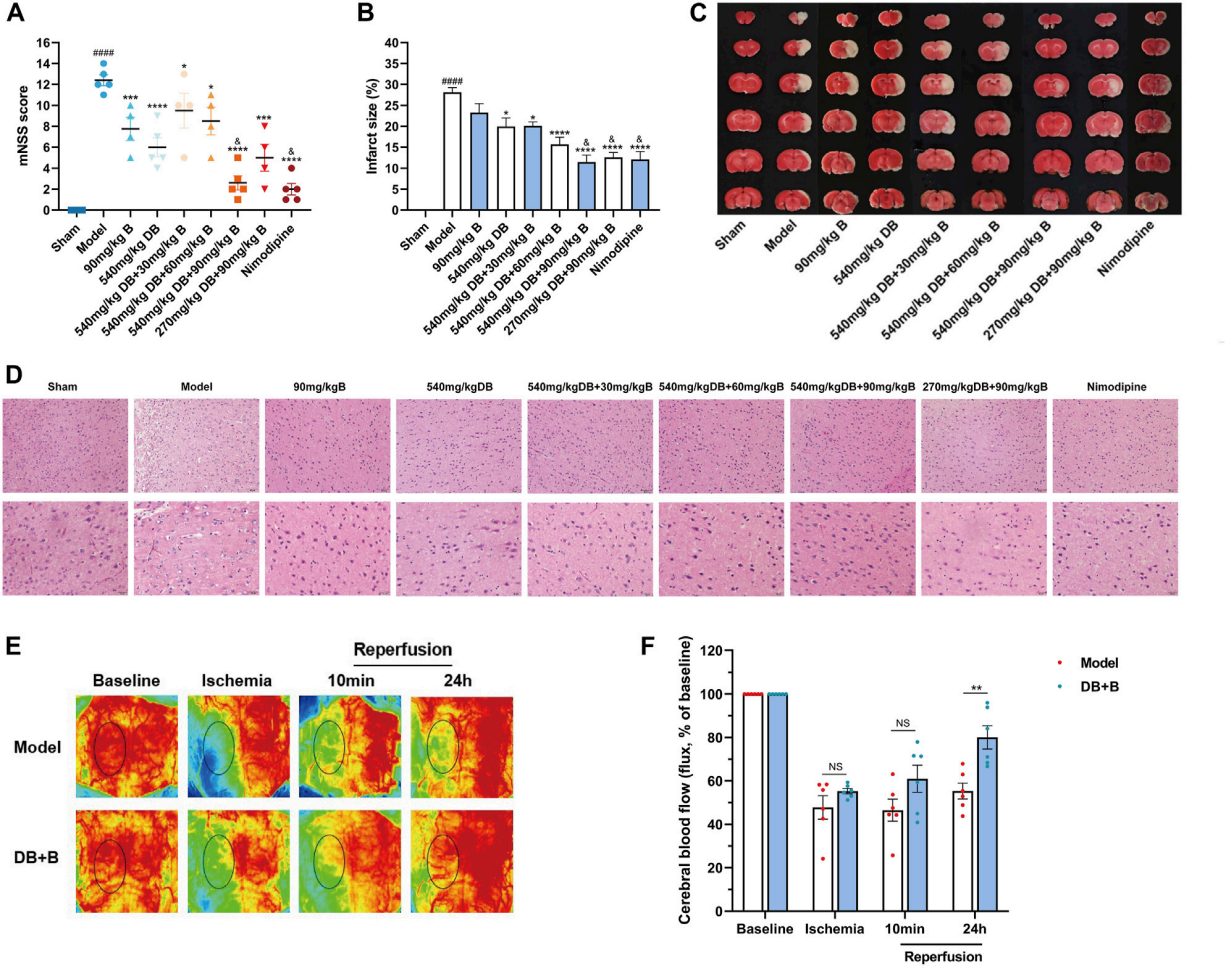
DB + B alleviates IR brain injury in tMCAO rats. **(A)** mNSS after 22 h of the ischemia-reperfusion (n ≥ 3). **(B, C)** The infarction volumes are shown by TTC staining (n ≥ 3). **(D)** The HE staining with scales of 50 μm and 20 µm (n ≥ 3). **(E, F)** Local cerebral blood flow of rats at baseline, after middle cerebral artery occlusion, and 10 min and 24 h post-reperfusion (n = 6). Results are expressed as means ± SEM. ^####^ *p* < 0.0001 compared with the sham group. *p < 0.05, **p < 0.01, ***p < 0.001, ****p < 0.0001 compared with the model group. ^&^ *p* < 0.05 compared with the DB group.

The neuroprotective effects of different proportional doses of DB + B on IR brain injury were detected using the mNSS and cerebral infarct size. Compared to the model group, the administration of DB, different proportions of DB + B, and nimodipine significantly decreased the neurological deficit scores (Figure 1A). The results showed that DB + B improved motor nerve function in tMCAO rats, and the effect of 540 mg/kg DB + 90 mg/kg B was similar to that of nimodipine. Furthermore, TTC staining showed that the administration of DB, different proportions of DB and B, and Nimodipine significantly reduced the brain infarct volume (Figures 1B, C) and that the 540 mg/kg DB + 90 mg/kg B group had the most visible effect on alleviating neurologic deficits and infarction volumes in tMCAO rats.

To evaluate neuronal loss histopathologically, brain tissue slices from rats were processed using HE staining. Compared with the sham group, HE staining showed necrosis and pyknosis of nerve cells, deep staining of nuclei, inflammatory cell infiltration, and gliosis in the model group (Figure 1D). The brain tissue of rats in the different drug delivery groups showed a reduction in inflammatory cell infiltration or gliosis and some improvements in neuronal necrosis and loss in HE staining, notwithstanding that it still had some impairment, but it was not as serious as the model group. Combined with the results of mNSS and TTC staining, the 540 mg/kg DB + 90 mg/kg B group showed the most significant therapeutic effect against focal cerebral IR in rats. A dose of 540 mg/kg DB + 90 mg/kg B was administered for all subsequent experiments.

Additionally, to determine whether the DB + B improved stroke outcome in tMCAO rats was associated with improvement of local cerebral blood flow, we assessed the laser speckle imaging at different time points (Figure 1E, F). Compared to the model group, regional cerebral blood flow significantly improved after 24 h of reperfusion in the DB + B group. In a word, DB + B can alleviate IR brain injury in tMCAO rats.

### 3.2 Analysis of differential expression genes (DEGs)

**FIGURE 2:**
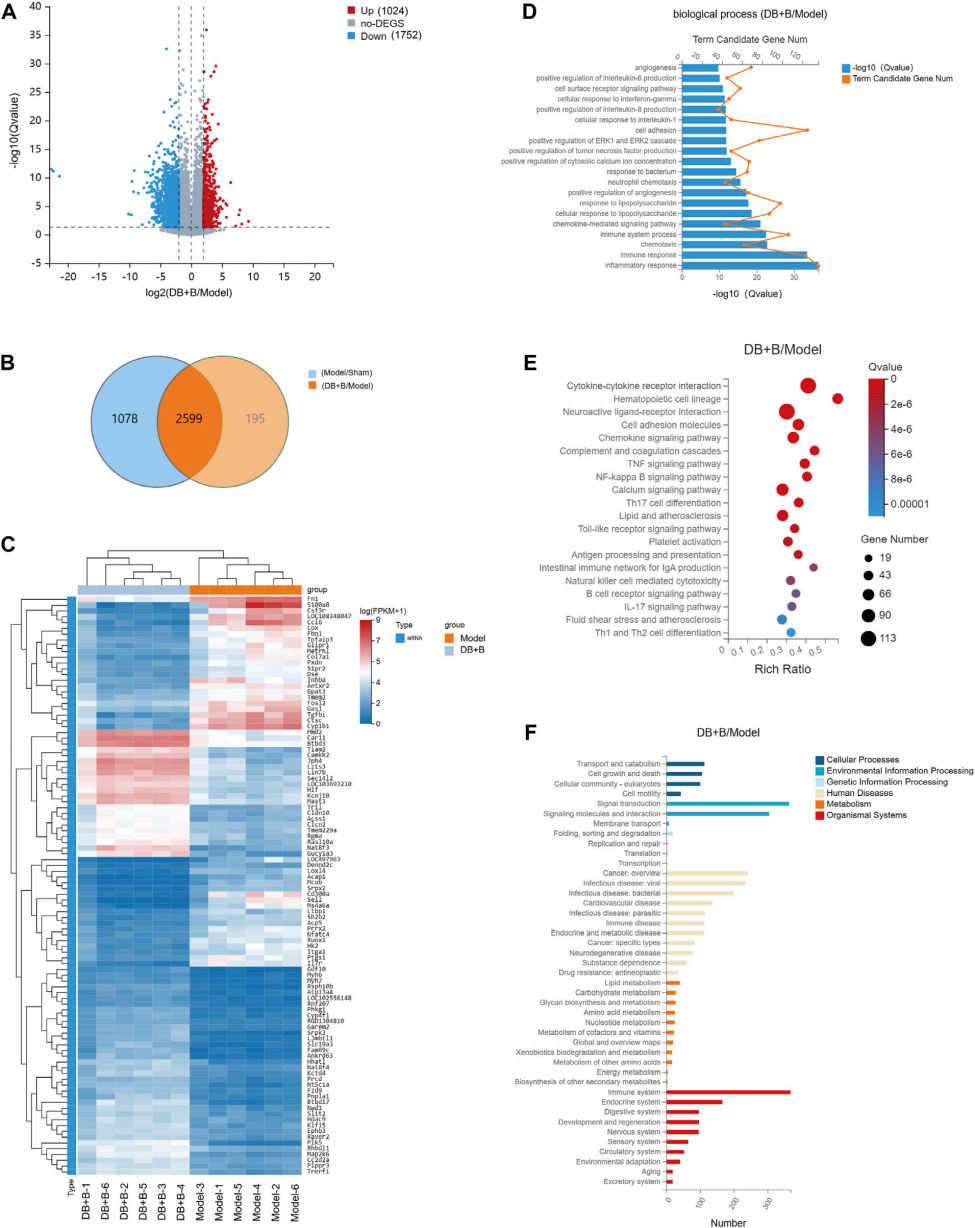
Analysis of DEGs. **(A)** The volcano plot of the DGEs between the model and DB + B group in the cerebral cortex of the ischemic hemisphere. **(B)** The VENN of DEGs between sham/model groups and DB + B/model groups. **(C)** Heat map of the DEGs between the model and DB + B group. **(D)** GO biological process enrichment analysis of DEGs between the model and DB + B group. **(E)** KEGG analysis of DEGs between the model and DB + B group. **(F)** The classification of the KEGG pathway in the DB + B/model group. n = 6.

RNA-sequencing was used to further explore the critical pathway for the neuroprotective effect of DB + B in IR brain injury. The significantly changed DEGs were clustering analyzed, and the thresholds of DEGs were set to log2FC ≥ 2, qvalue≤0.05. Approximately 3,677 genes differed significantly between the sham and model groups, of which 2,389 genes were upregulated and 1,288 genes were downregulated in the model group compared to the sham group (Supplementary Figure S1A). Meanwhile, 2,794 genes differed significantly between the DB + B and model groups, of these 1,024 genes were upregulated, and 1752 genes were downregulated in the DB + B group compared to the model group (Figure 2A). The VENN diagram shows the DEGs between the sham/model groups and DB + B/model groups (Figure 2B). We selected the top 100 significantly altered DEGs of the sham/model groups and DB + B/model groups, as shown in heatmaps (Figure 2C; Supplementary Figure S1B). Furthermore, we verified the results of RNA-Seq by the Western blot experiments of c-Jun and IL-6 (Supplementary Figures S1C–E).

GO is divided into biological processes, molecular functions, and cellular components. The DEGs were annotated using GO. According to GO functional enrichment analysis, it can be seen that the DEGs of the DB + B group compared to the model group are mainly concentrated in the biological processes of inflammatory response, immune response, immune system process, positive regulation of pro-inflammatory factor such as IL-6 and IL-8, chemokine-mediated signaling pathway, and so on (Figure 2D)). Similar results were found in the model group compared to the sham group (Supplementary Figure S1F).

According to KEGG analysis (Figure 2E; Supplementary Figure S1G), DB + B was found to play a role in cytokine-cytokine receptor interaction, hematopoietic cell lineage, neuroactive ligand-receptor interaction, cell adhesion molecules, chemokine signaling pathway, TNF signaling pathway, NF-κB signaling pathway, IL-17 signaling pathway, Th17 cell differentiation. Most were linked to the immune system, metabolism, signal transduction, nervous system, and infectious microbes (Figure 2F; Supplementary Figure S1H).

### 3.3 Analysis of differential metabolites

**FIGURE 3:**
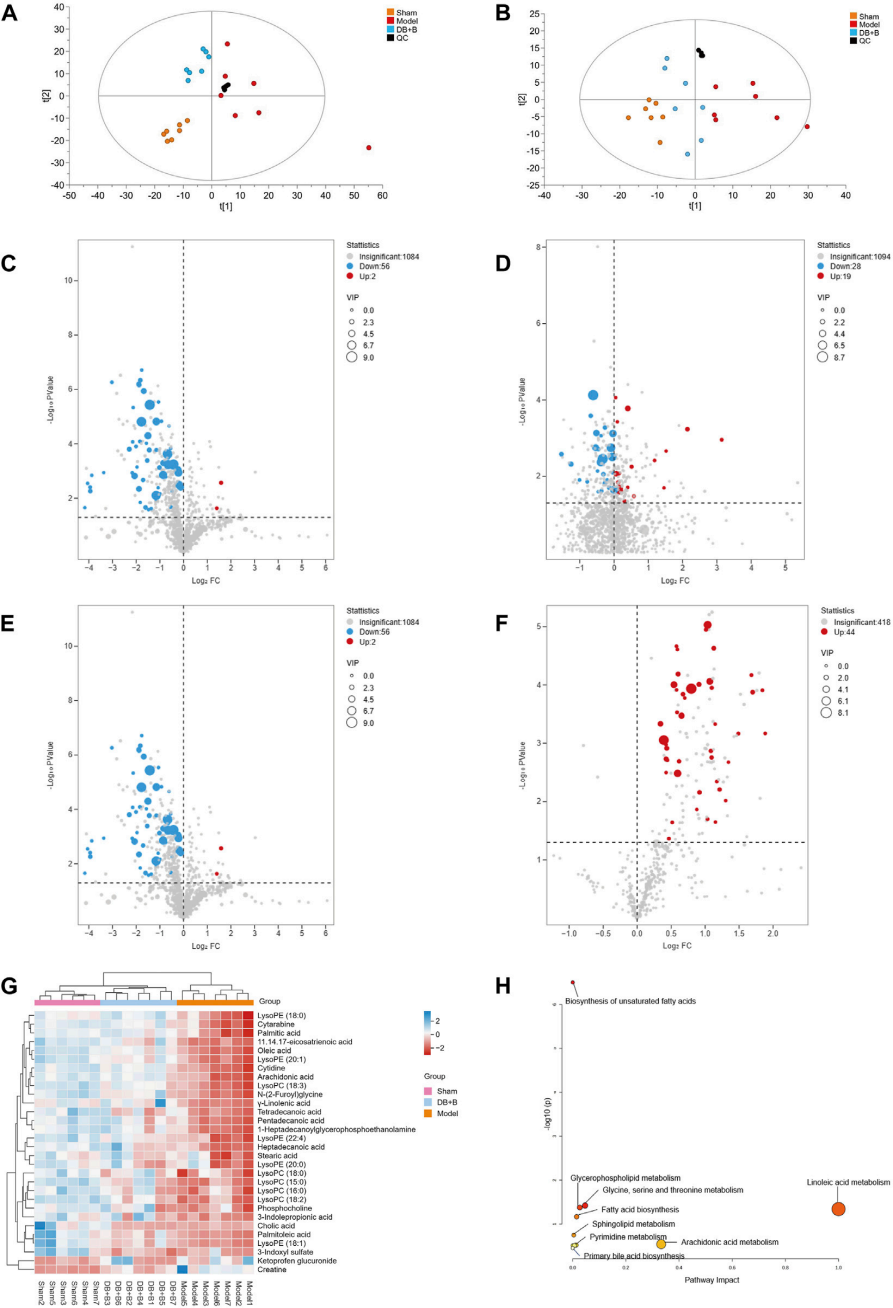
Analysis of differential metabolites. **(A)** PCA analysis of positive ion mode in the sham, model, and DB + B group. **(B, C)** The volcano plot of differential metabolites between the sham and model group in positive ion mode. **(D)** The volcano plot of differential metabolites between the model and DB + B group in positive ion mode. **(E)** The volcano plot of differential metabolites between the sham and model group in negative ion mode. **(F)** The volcano plot of differential metabolites between the model and DB + B group in negative ion mode. **(G)** Heat map of the differential metabolites in the sham, model, and DB + B group. **(H)** The pertinent metabolic pathway of differential metabolites. n = 7.

Serum metabolites were assessed by UPLC-Q/TOF-MS, and the total ion chromatograms of the three groups were separated well in both positive and negative ion modes within 21 min. First, PCA analysis of metabolomics demonstrated that the sham, model, and DB + B groups were distinct, and the DB + B group had a tendency to relocate to the sham group (Figure 3A, B). The OPLS-DA model was used to further analyze the metabolic differences between the sham, model and DB + B groups (Supplementary Figure S2A, C, E, G). In negative ion mode, the R^2^Y and Q^2^ values were 0.965 and 0.823 when the model group was compared to the sham group, and the R^2^Y and Q^2^ values were 0.925 and 0.809 when the DB + B group was compared to the sham group (Supplementary Figure S2B, D). In the positive ion mode, the R^2^Y and Q^2^ values were 0.958 and 0.882 when the model group was compared to the sham group, and the R^2^Y and Q^2^ values were 0.873 and 0.647 when the DB + B group was compared to the model group (Supplementary Figure S2F, H). Furthermore, the R^2^Y-intercepts and Q^2^-intercepts were lower than the original values, demonstrating the high predictivity and accuracy of fit of the established model.

Potential biomarkers in serum that helped to distinguish the model group from the sham group were selected with the following criterion: VIP ≥1 and *p* ≤ 0.05 (Figure 3C–F). A total of 30 endogenous metabolites with significant differences were identified using available biochemical databases (Table 3), the trend of these differential metabolites was shown in Figure 3G. MetaboAnalyst 5.0 was used to analyze the pertinent metabolic pathways of 40 potential biomarkers, including organic acids and derivatives, lysophospholipids, and organic heterocyclic compounds. The glycine, serine, and threonine metabolism; glycerophospholipid metabolism; arachidonic acid metabolism; linoleic acid metabolism; biosynthesis of unsaturated fatty acids; fatty acid biosynthesis; fatty acid elongation and degradation; pyrimidine metabolism; arginine and proline metabolism; and primary bile acid biosynthesis were found to be relevant to IR brain injury in rats (Figure 3H). In the presence of DB + B, 24 potential biomarkers were significantly regulated (*p* < 0.05), including creatine, 3-indolepropionic acid, lysoPC(15:0), lysoPC (18:2), lysoPC(18:0), tetradecanoic acid,γ-linolenic acid, palmitoleic acid, lysoPE (18:1), pentadecanoic acid, 1-heptadecanoylglycerophosphoethanolamine, lysoPC(18:3), N-(2-Furoyl)glycine, cytidine, lysoPC(22:4), arachidonic acid, cytarabine, lysoPE (18:0), 11.14.17-eicosatrienoic acid, oleic acid, palmitic acid, lysoPE (20:1), heptadecanoic acid, lysoPE (20:0).

**TABLE 3:** Identified potential biomarkers in the brain regulated by DB + B.

	Retention time (min)	m/z	Pseudo molecular ion	Formula	Metabolite name	HMDB	Change trend (M/S)	VIP (M/S)	Change trend (DB + B/M)	VIP (DB + B/M)
ESI+	1.019	132.0761	[M + H] +	C4H9N3O2	Creatine	HMDB0000064	↑##	2.00	↓*	1.59
	5.592	190.0857	[M + H] +	C11H11NO2	3-Indolepropionic acid	HMDB0002302	↓##	1.31	↑**	1.30
	9.920	482.3239	[M + H] +	C23H48NO7P	LysoPC (15:0)	HMDB0010381	↓##	4.00	↑**	2.84
	9.935	520.3386	[M + H] +	C26H50NO7P	LysoPC (18:2)	HMDB0010386	↓##	8.94	↑**	6.64
	10.573	184.07401	[M + H] +	C5H15NO4P+	Phosphocholine	HMDB0001565	↓##	1.01	NS	0.77
	10.573	496.33743	[M + H] +	C24H50NO7P	LysoPC (16:0)	HMDB0010382	↓##	8.28	NS	5.91
	13.063	524.3705	[M + H] +	C26H54NO7P	LysoPC (18:0)	HMDB0011128	↓##	6.52	↑*	5.49
ESI-	4.627	212.0020	[M−H] -	C8H7NO4S	3-Indoxyl sulfate	HMDB0000682	↓##	1.83	NS	0.02
	5.305	429.1954	[M−H] -	C22H22O9	Ketoprofen glucuronide	HMDB0010334	↑#	1.23	NS	0.57
	7.224	407.2795	[M−H] -	C24H40O5	Cholic acid	HMDB0000619	↓##	2.51	NS	0.42
	8.812	227.2015	[M−H] -	C14H28O2	Tetradecanoic acid	HMDB0000806	↓##	1.94	↑*	1.43
	9.334	277.2170	[M−H] -	C18H30O2	γ-Linolenic acid	HMDB0003073	↓##	1.02	↑**	1.29
	9.464	253.2170	[M−H] -	C16H30O2	Palmitoleic acid	HMDB0003229	↓##	2.59	↑**	2.02
	9.466	478.2932	[M−H] -	C23H46NO7P	LysoPE (18:1)	HMDB0011475	↓##	1.25	↑**	0.95
	9.852	241.2172	[M−H] -	C15H30O2	Pentadecanoic acid	HMDB0000826	↓##	2.00	↑**	1.99
	9.858	466.2939	[M−H] -	C22H46NO7P	1-Heptadecanoylglycer-phosphoethanolamine	HMDB0061691	↓##	1.07	↑**	1.10
	9.983	528.3085	[M + Na−2H] -	C26H48NO7P	LysoPC (18:3)	HMDB0010388	↓##	1.18	↑**	1.11
	10.237	168.0433	[M−H] -	C7H7NO4	N-(2-Furoyl)glycine	HMDB0000439	↓##	1.37	↑**	1.37
	10.253	242.0797	[M−H] -	C9H13N3O5	Cytidine	HMDB0000089	↓##	1.30	↑**	1.34
	10.273	528.3084	[M−H] -	C27H48NO7P	LysoPE (22:4)	HMDB0011493	↓##	2.85	↑**	2.59
	10.275	303.2324	[M−H] -	C20H32O2	Arachidonic acid	HMDB0001043	↓##	4.22	↑**	4.48
	10.502	255.2324	[M−H] -	C16H32O2	Palmitic acid	HMDB0000220	↓##	4.71	↑**	5.19
	10.887	224.0692	[M-H2O−H] -	C9H13N3O5	Cytarabine	HMDB0015122	↓##	2.27	↑**	2.51
	10.889	480.3088	[M−H] -	C23H48NO7P	LysoPE (18:0)	HMDB0011130	↓##	2.49	↑**	3.21
	10.977	305.2481	[M−H] -	C20H34O2	11.14.17-eicosatrienoic acid	HMDB0060039	↓##	2.51	↑**	2.27
	11.479	281.2480	[M−H] -	C18H34O2	Oleic acid	HMDB0000207	↓##	5.55	↑**	5.57
	11.482	506.3251	[M−H] -	C25H50NO7P	LysoPE (20:1)	HMDB0011482	↓##	2.69	↑**	2.59
	11.944	269.2481	[M−H] -	C17H34O2	Heptadecanoic acid	HMDB0002259	↓##	2.22	↑**	2.21
	12.610	508.3403	[M−H] -	C25H52NO7P	LysoPE (20:0)	HMDB0011482	↓##	1.48	↑**	1.33
	13.001	283.2633	[M−H] -	C18H36O2	Stearic acid	HMDB0000827	↓##	4.71	NS	5.14

LysoPC, lysophosphatidylcholine; lysoPE, lysophosphatidylethanolamine.

#*p* < 0.05.

##*p* < 0.01, vs sham group.

*p < 0.05.

**p < 0.01, vs model group; ↑ upregulated; ↓ downregulated.

### 3.4 Correlation analysis of DEGs and serum metabolites

**FIGURE 4:**
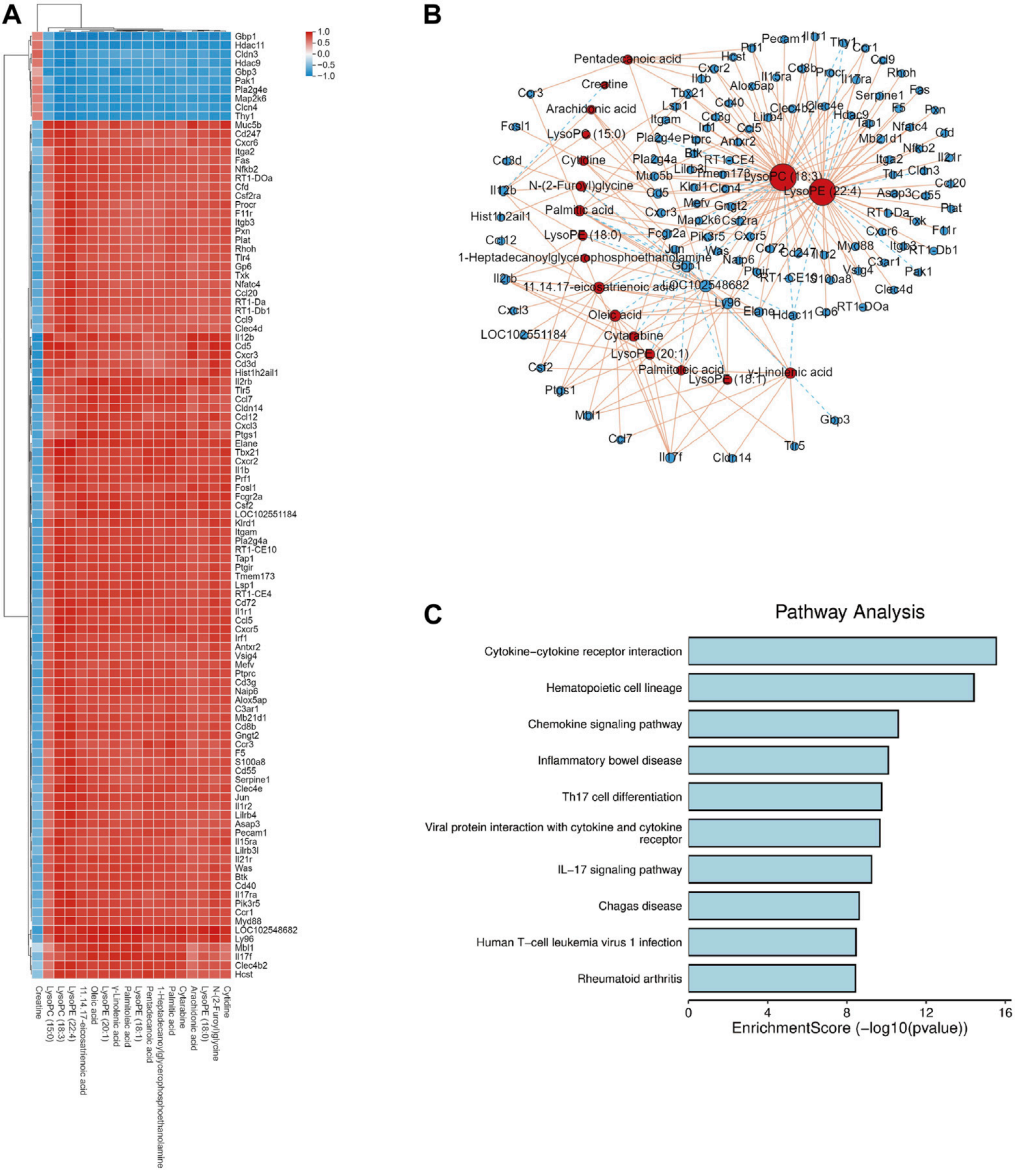
Correlation analysis of DEGs and serum metabolites. **(A)** Heat map of correlation between DEGs and serum metabolites. **(B)** Network diagram depicting DEGs (circles) and serum metabolites (triangles), and solid lines indicate positive correlation while dashed lines indicate negative correlation. **(C)** KEGG analysis of the DEGs which has significant correlations with serum metabolites.

In the KEGG analysis of DEGs, most of the pathways belonged to the immune system, so Spearman’s correlation analysis of DEGs in the immune system and metabolites was performed between the DB + B and model groups to further assess the molecule mechanism of DB + B in preventing IR brain injury. The clustered heatmap showed that the metabolites are mainly positively correlated with pro-inflammatory genes, including *IL-17f*, *Il-17ra*, *Jun*, *IL-6*, *Myd88*, and *Tlr4* (Figure 4A). We showed the correlation network graph between these DEGs and metabolites (Figure 4B), in which the lysoPC(18:3) and lysoPE (22:4) had the most significant correlations with most of the DEGs. Most of these DEGs belong to the IL-17 signing pathway (Figure 4C).

### 3.5 Ecological landscape of intestinal flora in tMCAO rats

**FIGURE 5:**
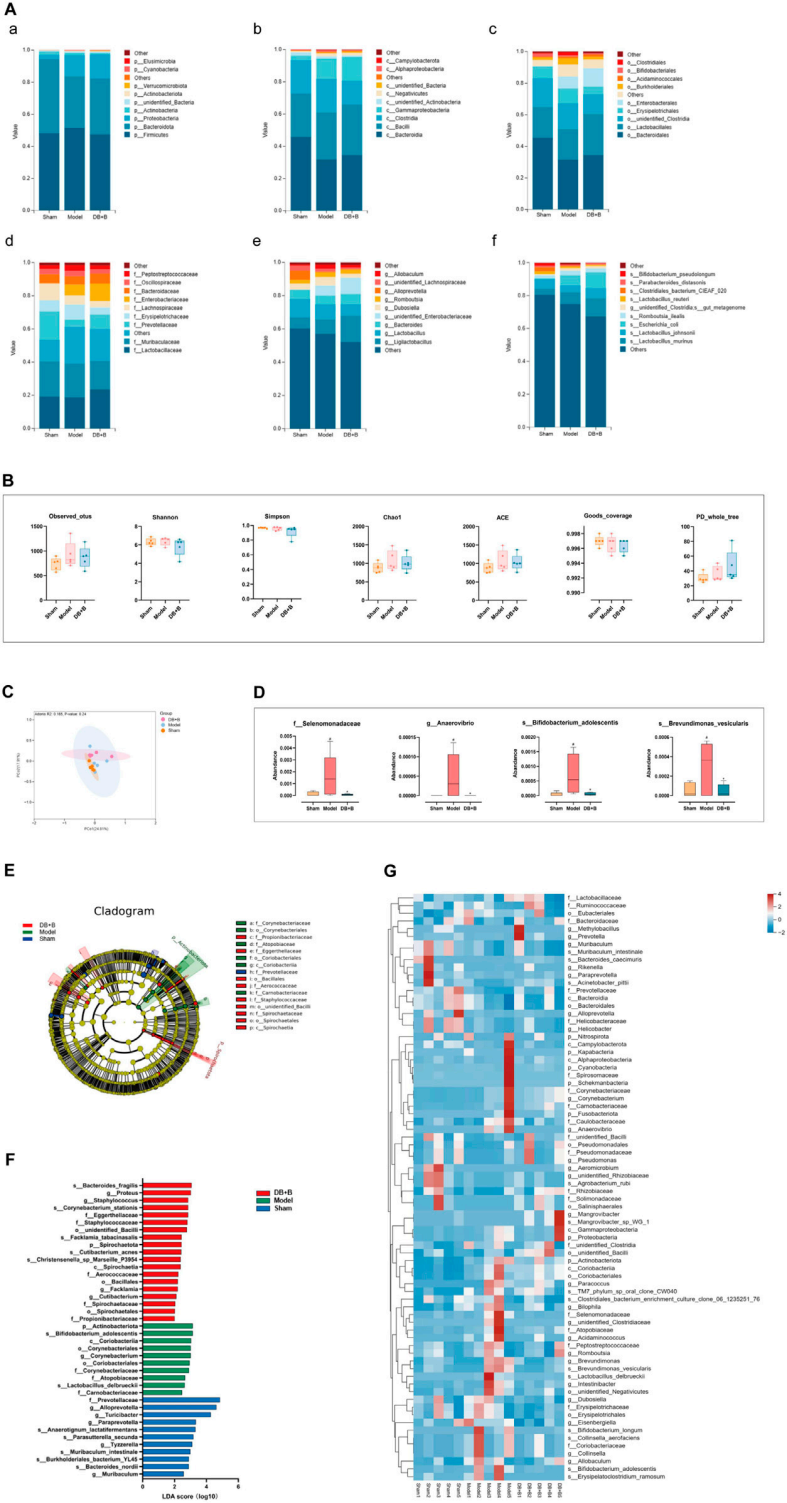
Ecological landscape of intestinal flora in tMCAO rats. **(A)** Histogram of relative abundances of species in each group and classification level, arranged from a to f as phylum, class, order, family, genus, and species. **(B)** Analysis of differences in α-diversity index between groups (from left to right: observed_otus, Shannon, Simpson, chao1, ace, good_coverage, and PD_whole_tree index). **(C)** The differences in β-diversity between groups using weighted Unifrac distance. **(D)** Metastas analysis between groups. **(E)** Evolutionary clade diagram. **(F)** LDA score. **(G)** Heatmap of differential gut microbiota between groups. n = 5. ^#^ *p* < 0.05, compared with the sham group. *p < 0.05, compared with the model group.

16S rRNA amplicon sequencing analysis was carried out to investigate the effect of DB + B on the dysbiosis of intestinal flora in tMCAO rats. We observed differences in the abundance of these groups at each classification level (i.e., phylum, class, order, family, genus, and species) when analyzing the species composition of the intestinal flora in the groups (shown in Figure 5A; the species composition of intestinal microbes in each sample is shown in Supplementary Figure S3A). The ɑ-diversity and β-diversity analysis was performed to detect significant differences in the intestinal flora within and among samples. The treatment of DB + B of IR brain injury had no apparent effect on ɑ-diversity (Figure 5B). PCoA indicated that the DB + B and model groups were partially separated (Figure 5C). Based on the metastasis analysis, *f_Selenomonadaceae*, *g_Anaerovibrio*, *s_Bifidobacterium_adolescentis*, and *s_Brevundimonas_vesicularis* were significantly altered in the intestinal flora (Figure 5D). Based on the linear discriminant analysis effect size, we found that the intestinal flora that differed significantly between the groups could be divided into 16 categories (Figure 5E). Statistically significant biomarkers between groups were species with an LDA value of >2 (Figure 5F). The t-test results showed that *f_Prevotellaceae*, *P_Actinobacteriota*, *g_Muribaculum*, and *s_Muribaculum_ intestinale* significantly changed between the model and sham groups (Supplementary Figure S3B), and *g_Eisenbergiella* and *o_unidentified_Bacilli* significantly changed between the DB + B and model groups (Supplementary Figure S3C). Additionally, the heat map shows the differential intestinal flora found by metastasis analysis, linear discriminant analysis effect size, and T-test (Figure 5G).

### 3.6 Correlation analysis of intestinal flora and serum metabolites

**FIGURE 6:**
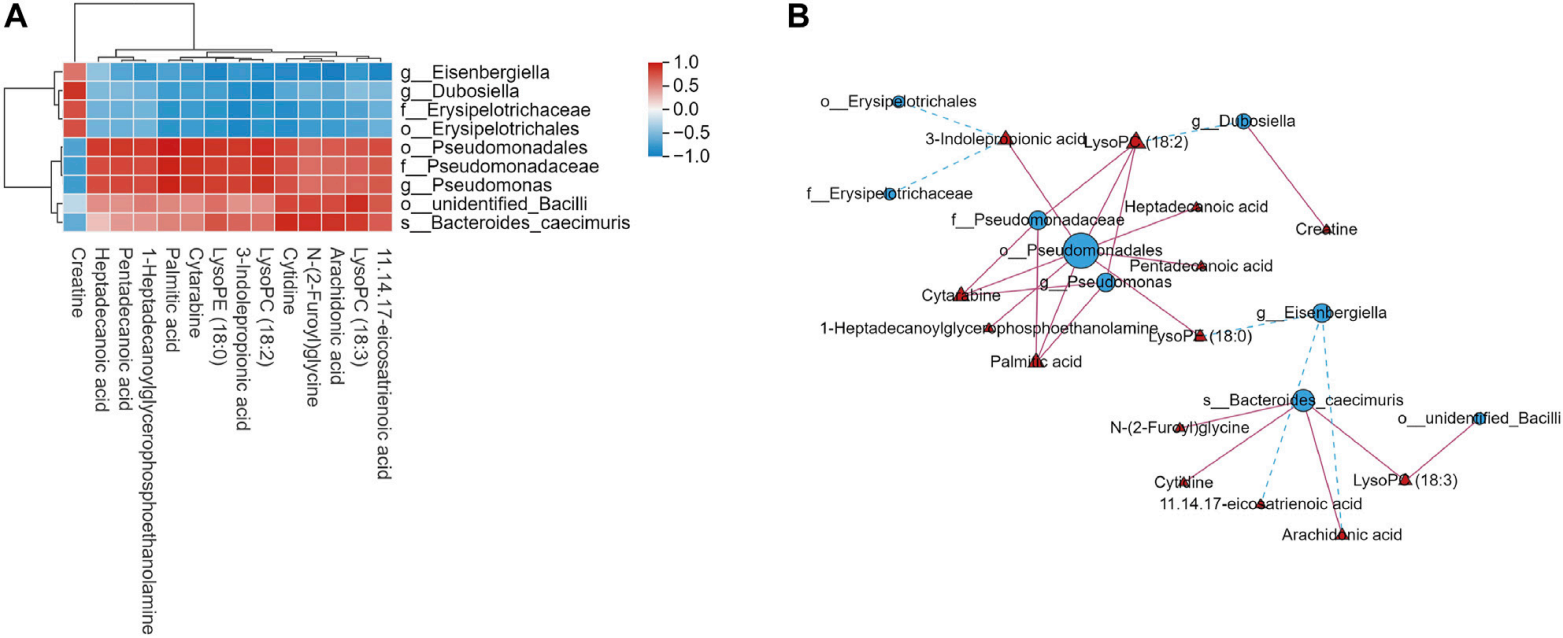
Correlation analysis of intestinal flora and serum metabolites. **(A)** Heat map of correlation between microbial diversity and serum metabolites. **(B)** Network diagram depicting intestinal flora (circles) and serum metabolites (triangles), and solid lines indicate positive correlation while dashed lines indicate negative correlation.

We found significant associations between serum metabolites and intestinal flora using Spearman’s correlation analysis between the DB + B and model groups (Figure 6A). lysoPC(18:3) N-(2-Furoyl)glycine and cytidine are positively correlated with *o_unidentified_Bacilli* and *s_Bacteroides_caecimuris*. LysoPC(18:1), lysoPE (18:0), palmitic acid, cytarabine, and 3-indole propionic acid are positively correlated with *o_Pseudomonadales*, *f-Pseudomonadaceae*, and *g-Pseudomonas*. The network graph showed the correlation between these flora and metabolites (Figure 6B). *o_Pseudomonadales* and *s_Bacteroides_caecimuris* had the most significant correlations with the most serum metabolites. The results indicated that the DB + B improvement in IR brain injury was associated with changes in the intestinal flora and its metabolites, and *o_Pseudomonadales* and *s_Bacteroides_caecimuris* are the most important intestinal flora.

## 4 Discussion

In this study, we found for the first time that DB + B enhanced the efficacy of ischemic stroke therapy compared to DB or B alone. As the increases dose of DB and B in different proportional doses of DB + B groups, the treatment of IR brain injury in rats is more effective. After DB + B treatment, motor nerve function was significantly improved, infarct area and neuronal necrosis and loss were significantly reduced, and regional cerebral blood flow significantly improved.

The enrichment analysis showed that the DEGs were mainly related to the biological immune response, metabolism, signal transduction, nervous system, and microbial infection. Consistent with these results, cytokine-cytokine receptor interactions involve a variety of immune-related pathways. Neuroactive ligand-receptor interactions are related to ferroptosis in vascular neurons (Zhang et al., 2022). NF-κB signaling pathways and Th 17 cell differentiation were significant in inflammatory response following ischemic stroke (Zhang et al., 2016). TNF signaling pathways may play a significant role in the treatment of stroke and traumatic brain injury (Bruce et al., 1996). IL-17 is a pro-inflammatory cytokine mainly produced by γδ T cells and Th 17 cells, including six members (IL-17A to IL-17F), which have an important role in neurological disorders. Abnormal production of IL-17 can lead to excessive pro-inflammatory cytokine production and chronic inflammation, resulting in tissue damage (Qian et al., 2007; Gaffen, 2009; Majumder et al., 2019). Furthermore, we verified the expression of c-Jun and IL-6 through Western blot, because c-Jun and IL-6 belong to IL-17 sigining pathway and positive regulation of IL-6 production, and they have the significant correlations with serum metabolites.

These findings further support that the efficacy of DB + B for the prevention and treatment of IR brain injury is closely linked to the immune response and signal transduction.

Abnormal metabolic changes in the internal biochemical environment are often caused by this disease. Due to the impairment of the blood-brain barrier, differential metabolites that may be potential biomarkers of ischemic stroke can be distinguished in the serum (Montaner et al., 2020). The inflammatory response in ischemic stroke affects phospholipid metabolism, resulting in perturbed metabolism of lysoPCs and LysoPEs (Wang et al., 2005; Sidorov et al., 2019). LysoPCs and lysoPEs are products of phospholipase A2 activity, and play an important role in toxic inflammatory responses in a wide range of organ systems (Cunningham et al., 2008). The efficacy of lysoPCs against cerebral ischemia and glutamate excitotoxicity has been demonstrated in previous studies (Blondeau et al., 2002). Our results showed that DB + B increased the expression of lysoPC (15:0), lysoPC (18:2), lysoPC (18:0), lysoPC (18:3), lysoPC (22:4), lysoPE (18:1), lysoPE (18:0), lysoPE (20:1), and lysoPE (20:0) in rats with ischemia-reperfusion injury. Essential for normal human growth, development, and physiological function, polyunsaturated fatty acids have immunomodulatory effects as well as the ability to modulate inflammation, neurotransmission, and vascular reactivity (Poorani et al., 2016). The γ-linolenic acid is the precursor of arachidonic acid (Poorani et al., 2016), and some studies have shown that platelet arachidonic acid levels were significantly reduced in stroke patients and that acetylsalicylic acid could normalize the arachidonic acid values in the platelet (Färkkilä et al., 1987). Arachidonic acid can be converted into lipoxin A4, which inhibits the expression of inflammatory cytokines including NF-κB and lipocalin (Gundala et al., 2017). Arachidonic acid is vital for the structure and function of vascular membranes. The ɑ-linolenic acid is the precursor of docosahexaenoic acid and eicosapentaenoic acid, which produce both pro-inflammatory and anti-inflammatory metabolites (Das, 2021). DB + B normalized the levels of polyunsaturated fatty acids such as arachidonic acid, γ-linolenic acid, tetradecanoic acid, palmitoleic acid, pentadecanoic acid, 11.14.17-eicosatrienoic acid, oleic acid, and heptadecanoic acid, in rats with ischemic stroke. Creatine is an amino acid found naturally in human tissues. It is synthesized mainly in the liver and kidney, transported through the blood, and absorbed by tissues with high energy requirements, such as the brain (Wallimann et al., 1992). Thus, under the pathological conditions of ischemic stroke, creatine may accumulate in the serum. Additionally, 3-indole propionic acid, produced from tryptophan degradation, was increased in the DB + B group and is known to have biological activities, such as antioxidation and resistance to inflammation (Smith and Macfarlane, 1996). Furthermore, 1-Heptadecanoylglycerophosphoethanolamine, N-(2-Furoyl)glycine, cytidine, cytarabine, and sclareol were identified as potential biomarkers for ischemic stroke in our study.

In our analysis, most of the DEGs and abnormal metabolites were significantly associated with immunity and inflammation. Furthermore, some of the metabolites in our study were related to the intestinal flora, including phospholipids, 3-indole propionic acid, and pentadecanoic acid (Haase et al., 2020). Currently, a typical method for investigating suspected metabolic disorders and drug mechanisms involves the combination of 16S rRNA amplicon sequencing of microbial diversity with untargeted metabolomics. Thus, we performed further analyses to investigate the effects of the intestinal flora on the mechanism of DB + B in the amelioration of cerebral IR brain injury.

Based on the 16S rRNA amplicon sequencing data, we found that both the abundance and diversity of the intestinal flora recovered in the DB + B group compared with those in the model group. Pterostilbene (a phenolic metabolite in DB) has anti-arthritic effects (Rui et al., 2022), prevents steatohepatitis (Milton-Laskibar et al., 2021), and decreases vascular inflammation (Koh et al., 2019) by suppressing inflammation and altering intestinal bacteria. However, the effects of DB and its phenolic metabolites on the gut-brain axis have not been studied. Many studies have focused on the role of the intestinal flora in nervous system diseases. Human intestinal flora consists mainly of *Bacteroidetes* and *Firmicutes*, with the remainder consisting of *Actinobacteria*, *Verrucomicrobia*, and *Proteobacteria* (Eckburg et al., 2005). Dysbiosis following stroke is characterized by altered *Firmicutes*, *Bacteroidetes*, and *Actinobacteria* (Sorboni et al., 2022). However, in our study, DB + B alleviated cerebral IR brain injury by regulating the abundance of *Cyanobacteria*, *Fusobacteriota*, *Kapabacteria*, *Nitrospirota*, and *Schekmanbacteria* apart from *Actinobacteriota* and *Proteobacteria*. Another study suggested that patients with transient ischemic attack and stroke had a decreased number of beneficial or commensal genera, including *Bacteroides* and *Prevotella* (Yin et al., 2015). Cognitive impairment after a stroke is associated with an abundance of *Clostridia*, *Lactobacillus*, and *Prevotella* (Ling et al., 2020). DB + B reversed the changes in the intestinal flora in tMCAO rats. Additionally, our results showed that *o_unidentified_Bacilli, f_Selenomonadaceae*, *g_Anaerovibrio*, *s_Bifidobacterium_adolescentis*, *g_Eisenbergiella, g_Muribaculum*, *s_Brevundimonas_vesicularis*, and *s_Muribaculum_intestinale* were significantly regulated by DB + B. It was shown that intestinal flora is an intermediary of the protective effect of DB + B in IR brain injury.

To further investigate the protective effect of DB + B in IR brain injury, we performed Spearman’s correlation analysis between DEGs, serum metabolites, and intestinal flora. In our results, the serum metabolites are mainly positively correlated with pro-inflammatory genes, including *IL-17f*, *Il-17ra*, *IL-6*, *Myd88*, and *Tlr4*, and the lysoPC(18:3) and lysoPE (22:4) had the most significant correlations with IL-17 signing pathway. And *o_Pseudomonadales* and *s_Bacteroides_caecimuris* have had the most significant correlations with the most serum metabolites. A study has shown that intestinal bacterial species emerge to modulate the neuroinflammation after a stroke by regulating intestinal T-cell infiltration into the brain because antibiotic-induced changes in intestinal flora led to a decrease in the expression of IL-17-associated chemokine and decreased migration of pro-inflammatory γδ T cells (Benakis et al., 2016). Ginsenoside Rg3 can inhibit the TLR4/MyD88/NF-κB pathway and regulate intestinal flora to downregulate the expression of proinflammatory factors (Duan et al., 2023). It is worth noting that our analysis also found that the IL-17 signaling pathway and NF-κB signaling pathway were significantly enriched in the cortex. Thus, our study indicates that the gut-brain axis plays an important role in the prevention of DB + B in IR injury.

Nevertheless, some limitations must be considered in our results. First, we performed transcriptomic analysis, non-targeted metabolomics, and 16S rRNA analysis, but did not further investigate the related pathways. Second, we only studied the preventive effect of DB + B on IR brain injury during the acute period. Therefore, we did not investigate the therapeutic effects of DB + B. Finally, the pathological changes in the hippocampus are worth exploring. Further studies are warranted.

## 5 Conclusion

In conclusion, our study provisionally confirms that DB + B can improve the protective benefit of IR brain injury by effectively regulating intestinal flora including *o_Pseudomonadales*, *s_Bacteroides_caecimuris*, *o_unidentified_Bacilli*, *f-Pseudomonadaceae*, and *g-Pseudomonas*, mainly regulating serum metabolites including lysoPCs and lysoPEs thus inhibiting TLR4/MyD88/NF-κB and IL-17 signing pathway to reduce inflammatory reactions. This scientifically gives support for the clinical translation of DB + B in the prevention and treatment of ischemic stroke and establishes a basis for further investigation of its therapeutic mechanism.

## Data Availability

The raw sequence data of RNA-sequencing reported in this paper have been deposited in the Genome Sequence Archive (Genomics, Proteomics & Bioinformatics 2021) in National Genomics Data Center (Nucleic Acids Res 2022), China National Center for Bioinformation/Beijing Institute of Genomics, Chinese Academy of Sciences (GSA: CRA014412) that are accessible at https://ngdc.cncb.ac.cn/gsa/search?searchTerm=CRA014412. The raw sequence data of 16S rRNA reported in this paper have been deposited in the NCBI (PRJNA1061643) that are accessible https://dataview.ncbi.nlm.nih.gov/object/PRJNA1061643?reviewer=5ehgmsu04klbgdcnimbkd8gc3.
