# Supplementary material for: Comprehensive evaluation of Dragon’s Blood in combination with borneol in ameliorating ischemic/reperfusion brain injury using RNA sequencing, metabolomics, and 16S rRNA sequencing
Source: Front Pharmacol. 2024 May 9;15:1372449. doi: 10.3389/fphar.2024.1372449 (PMC11112420; doi:10.3389/fphar.2024.1372449)
Supplement: Supplementary file 1 [file DataSheet1.docx]

**Supplementary Materials**

Table S1 Modified Neurological Severity Score Points

| Motor tests |  | Score |
| --- | --- | --- |
| Raising rat by tail | Flexion of forelimb | 1 |
|  | Flexion of hindlimb | 1 |
|  | Head moved >10° to vertical axis within 30 s | 1 |
| Placing rat on floor | Normal walk | 0 |
|  | Inability to walk straight | 1 |
|  | Circling toward paretic side | 2 |
|  | Falls down to paretic side | 3 |
| Sensory tests | Placing test (visual and tactile test) | 1 |
|  | Proprioceptive test (deep sensation, pushing paw against table edge to stimulate limb  muscles) | 1 |
| Beam balance tests | Balances with steady posture | 0 |
|  | Grasps side of beam | 1 |
|  | Hugs beam and 1 limb falls down from beam | 2 |
|  | Hugs beam and 2 limbs fall down from beam, or spins on beam (>60 s) | 3 |
|  | Attempts to balance on beam but falls off (>40 s) | 4 |
|  | Attempts to balance on beam but falls off (>20 s) | 5 |
|  | Falls off; no attempt to balance or hang on to beam (<20 s) | 6 |
| Reflex absence and abnormal movements | Pinna reflex (head shake when auditory meatus is touched) | 1 |
|  | Corneal reflex (eye blink when cornea is lightly touched with cotton) | 1 |
|  | Startle reflex (motor response to a brief noise from snapping a clipboard paper) | 1 |
|  | Seizures, myoclonus, myodystony | 1 |
| Maximum points |  | 18 |





Supplementary Figure 1. Analysis of DEGs. (A) The volcano plot of the DEGs between the sham and model group in the cerebral cortex of the ischemic hemisphere. (B) Heat map of the DEGs between the sham and model group. (A) GO molecular function enrichment analysis of DEGs between the sham group and the model group. (C) The image of Western blot. (D) Expression of c-Jun in cortex. (E) Expression of IL-6 in cortex. (F) GO biological process enrichment analysis of DEGs between the sham and model group. (G) KEGG analysis of DEGs between the sham and model group. (H) The classification of KEGG pathway in the model/sham group. n=6.



Supplementary Figure 2. Analysis of differential metabolites. (A) OPLS-DA analysis of positive ion mode in the sham group and model group. (B) The random model of positive ion mode in the sham group and model group. (C) OPLS-DA analysis of positive ion mode in the model group and DB+B group. (D) The random model of positive ion mode in the model group and DB+B group. (E) OPLS-DA analysis of negative ion mode in the sham group and model group. (F) The random model of negative ion mode in the sham group and model group. (G) OPLS-DA analysis of negative ion mode in the model group and DB+B group. (H) The random model of negative ion mode in the model group and DB+B group. n=7.


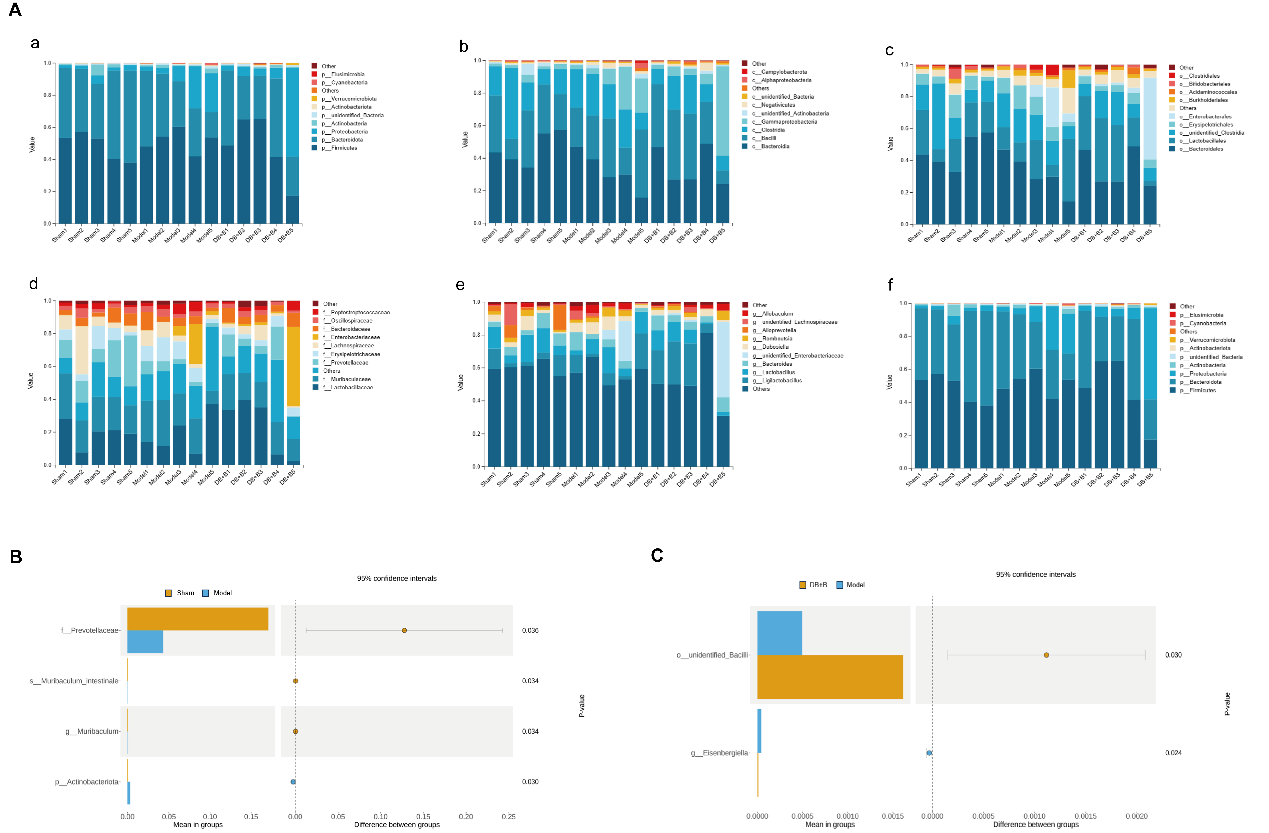


Supplementary Figure 3. Ecological landscape of gut microbes in tMCAO rats. (A) Histogram of relative abundances of species in each sample and classification level arranged as phylum, class, order, family, genus, and species from a to f. (B) T-test of differential gut microbiota between model group and sham group. (C) T-test of differential gut microbiota between DB+B group and model group.
